# Exploring the Physicochemical Compatibility of Minoxidil in Combination with Different Active Pharmaceutical Ingredients in Ready-to-use Vehicles for Alopecia Treatment

**DOI:** 10.2174/0115672018327249241217163930

**Published:** 2025-01-22

**Authors:** Bruna Marianni, Savvas Koulouridas, Hudson Caetano Polonini

**Affiliations:** 1 Global R&D Management, Fagron BV - Fascinatio Boulevard 350, 3065 - WB Rotterdam, The Netherlands

**Keywords:** Alopecia, personalized medicine, dermatology, medical treatment, chemical analysis, compatibility study, beyond-use date

## Abstract

**Background:**

Alopecia is globally known as a distressing medical disorder that affects men and women, and current commercially available minoxidil solutions are formulated with irritant vehicles with frequent complaints of dermatologic adverse effects.

**Objectives:**

This study aimed to investigate further the compatibility of ready-to-use vehicles for the preparation of tailored formulations for alopecia treatment, namely TrichoSol™ (a ready-to-use vehicle for personalized hair solutions) and TrichoFoam™ (a ready-to-use vehicle for personalized foam formulations), in combination with minoxidil and other active pharmaceutical ingredients (APIs), to establish adequate beyond-use dates (BUD) for the given formulations.

**Methods:**

Products under evaluation were compounded using TrichoSol™ or TrichoFoam™, with direct incorporation of the APIs into these vehicles. Samples were then stored at controlled room temperature for up to 180 days. High-performance liquid chromatography (HPLC) methods were developed and validated, and then utilized to evaluate the compatibility of the APIs in TrichoSol™ and TrichoFoam™. Forced degradation studies were conducted to assess API stability under various stress conditions, and Antimicrobial Effectiveness Testing (AET) was performed at 0 and 180 days after compounding.

**Results:**

According to our results, BUDs of up to 90-180 days were obtained for the examined formulations stored at room temperature, considering a degradation of maximum 10% of the nominal concentration of the APIs within them. The formulations exhibited no discernible physical alterations throughout this period and maintained chemical stability within acceptable limits. Microbiological evaluations confirmed the efficacy of the preservative system.

**Conclusion:**

Products compounded with TrichoSol™ and TrichoFoam™ showed suitable stability to be used as personalized treatments for alopecia. We can then suggest that the vehicles TrichoSol™ and TrichoFoam™ present effective solutions for compounding personalized hair care treatments.

## 1 INTRODUCTION

Alopecia is globally known as a distressing medical disorder that affects men and women, with noticeable psychosocial impacts and loss of self-confidence, potentially leading to anxiety and depression [1-4]. A careful clinical evaluation is paramount to determine the type of alopecia and its severity to elaborate an effective treatment protocol for each patient. The types of alopecia can be defined as: androgenetic alopecia (AGA), alopecia areata, telogen effluvium, cicatricial alopecia, and traction alopecia [5-10].

The Food and Drug Administration (FDA) has approved treatments such as topical minoxidil, oral finasteride, and low-level light therapy. Nevertheless, healthcare providers frequently employ a plethora of alternative primary and adjunctive approaches [11-13].

Minoxidil was initially discovered in the ’70s as a vasodilator drug, being an important part of antihypertensive treatment regimens, but the noticeable discontinuation of treatments from female patients due to the development of hirsutism led to further investigations into minoxidil potential effects for trichology [14, 15]. In the late 90s, the drug was approved by the FDA as a first-line topical treatment for androgenetic alopecia, and it continues to be so until the present day [16, 17]. The current commercially available minoxidil solutions are formulated with vehicles such as water, alcohol, and propylene glycol, and there is a frequent complaint of dermatologic adverse effects such as irritant contact dermatitis, and allergic contact dermatitis, causing pruritus, erythema, scaling and dryness of the scalp. Previous studies have shown that, most commonly, these adverse effects are triggered by propylene glycol [18, 19].

Given the negative impact of adverse reactions on patients’ compliance with long-term treatments, tailor-made formulations offer a potential opportunity to improve treatment efficacy through alternative vehicles and broaden the association possibilities. Additionally, there is growing interest in combining multiple therapeutic approaches, and literature supporting stability and compatibility data between the combinations is scarce.

In this study, we aimed to investigate further the physicochemical compatibility of ready-to-use vehicles for the preparation of tailored formulations for alopecia treatment, namely TrichoSol™ (a ready-to-use vehicle for personalized hair solutions consisting of a highly spreadable hydrophilic solution containing mineral salts of vegetable origin, free from alcohol and propylene glycol), and TrichoFoam™ (a ready-to-use vehicle for personalized foam formulations containing mineral salts of vegetable origin, free from propelants, alcohol and propylene glycol), in combination with minoxidil and other active pharmaceutical ingredients (APIs). Both TrichoSol™ and TrichoFoam™ contain in their composition TrichoTech™ - a patented phytocomplex with selected essential oils that can enhance their beneficial effects on the scalp [20]. In previous studies, these vehicles have been demonstrated to be compatible with an important range of APIs [21-24], filling important gaps in personalized alopecia treatments. In this sense, the 5 chosen formulations for this study were minoxidil combined with 17-alpha estradiol, clobetasol, dutasteride, finasteride, and ketoconazole, in bracketed concentrations based on the lowest and highest commonly prescribed formulations.17-alpha estradiol is a weaker estrogen that counteracts androgen activity by inhibiting the conversion of testosterone to dihydrotestosterone (DHT), a key factor in AGA. Clinical evidence supports its ability to slow the progression of hair loss, particularly in the early stages [25, 26]. Clobetasol, a potent corticosteroid, is widely used to suppress inflammation and immune responses, offering benefits in conditions like alopecia areata that can overlap with or mimic AGA [27]. Dutasteride and finasteride are 5-alpha reductase inhibitors that reduce DHT levels in the scalp, targeting the hormonal drivers of follicular miniaturization. While dutasteride inhibits both type I and type II 5-alpha reductase enzymes, finasteride selectively inhibits type II, offering complementary options for androgen suppression [28-30]. Ketoconazole, an antifungal agent with additional anti-androgenic and anti-inflammatory properties, is frequently included in AGA treatment regimens to address scalp conditions such as seborrheic dermatitis and enhance hair density [31, 32].

These APIs were selected for this compatibility study because of their strong clinical evidence, diverse mechanisms of action, and their potential to address multiple aspects of alopecia treatment, especially in combination with minoxidil.

This research seeks to explore effective and well-tolerated topical treatments for alopecia by determining validated beyond-use dates (BUDs) that ensure the safe preparation of customized therapies. The United States Pharmacopeia (USP) defines BUDs as the period within which a compounded product should be used to guarantee its physicochemical stability. Notably, USP guidelines offer general BUD recommendations when specific stability data are unavailable. Consequently, pharmacists must evaluate whether the available stability evidence warrants a shorter BUD than the general guideline, especially for APIs susceptible to degradation [33]. This underscores the importance of studies like this one in establishing appropriate BUDs.

## 2 MATERIALS AND METHODS

### 2.1 Equipment

The high-performance liquid chromatography (HPLC) analyses were conducted using a validated and calibrated chromatography system from Young Lin (Anyang, Korea). The system consisted of the following components: a quaternary pump (YL 9110), a photodiode array detector (YL 9160), an automatic injector (YL 9150), a column compartment (YL 9131), and a software controller (Clarity).

### 2.2 Reagents, Reference Standards, and Materials

The raw powders of all APIs (Table **1**) and the vehicles TrichoSol™ and TrichoFoam™ were provided by Fagron (chosen concentrations were based on common clinical/dermatological practice worldwide). The concentrations and intended purposes of the APIs are provided in Table **1**. For the HPLC analyses, high-performance liquid chromatography-grade reagents from Panreac (Barcelona, Spain) were employed. Ultrapure water, obtained from the AquaMax-Ultra 370 Series (Young Lin, Anyang, Korea), with a resistivity of 18.2 MΩ-cm at 25 °C, was utilized throughout the experiments. The reference standards used were sourced from the official primary materials of the USP (Rockville, MD, USA). To ensure purity, the mobile phases and receptor media were filtered using a 0.45-µm filter membrane (RC-45/15 MS; Chromafil, Düren, Germany) and degassed using an ultrasonic apparatus (Model 1600A; Unique, Indaiatuba, Brazil) for 30 minutes before use. All volumetric glassware and the analytical balance employed were duly calibrated.

### 2.3 Chromatographic Determinations

The analytical conditions employed for each API are provided in Table **2**. Those methods were developed in-house and then validated. The APIs were assayed separately, even when in the same combination. For consistency, each column was linked to a pre-column with identical specifications from the same supplier.

### 2.4 Validation of HPLC Method for Forced Degradation Studies: Assessing Stability Indicators

To validate the capability of the HPLC method in detecting potential degradation products during sample storage, the API samples were subjected to various stressing conditions (Tables **3**. and **4**.). These conditions included dilution in 0.1 M HCl, 0.1 M NaOH, and hydrogen peroxide (H_2_O_2_), exposure to ultraviolet light at 365 nm for 24 hours, and heating at 70 °C for 24 hours.

Following the study, all solutions were analyzed using HPLC, and any additional peaks observed in the chromatograms were identified and labeled. The resolution between * Gradient: 0.0 - 1.0 min, SB 100%; 1.0 - 1.1 min, SA 10%, SB 90%; 1.1 - 2.0 min, SA 30%, SB 70%; 2.0 - 3.0 min, SA 40%, SB 60%; 3.0 - 4.0 min, SA 70%, SB 30%; 4.0 - 5.0 min, SA 100%; 5.0 - 5.5 min, SA 100%; 5.5 - 5.6 min, SA 90%, SB 10%; 5.6 - 5.7 min, SA 70%, SB 30%; 5.7 - 5.8 min, SA 40%, SB 60%; 5.8 - 5.9 min, SA 20%, SB 80%; 5.9 - 6.0 min, SA 10%, SB 90%; 6.0 - 7.0 min, SB 100%; 7.0 - 9.0 min, SB 100%; 9.0 - 10.0 min, SB 100%.

the degradant and the API was determined, with a resolution of 1.5 or higher between peaks considered as complete separation. The study adhered to the recommendations outlined in the International Council for Harmonization of Technical Requirements for Pharmaceuticals for Human Use (ICH) Guideline Q1A(R2) on Stability Testing of New Drug Substances and Products [36]. These stressing conditions encompassed temperature, humidity, oxidation, and photolysis effects on the drug substance. Additionally, the study evaluated the drug substance's susceptibility to hydrolysis across a wide range of pH values in solution or suspension. Photostability testing was also incorporated as an essential component of the stress testing process.

The HPLC methods mentioned in this study were validated following internal protocols that were already used for other works from our research group [37], aligned with the guidelines provided by the USP and the ICH [38-40].

To assess the specificity of each method, comparisons were made using HPLC analyses of a standard solution, a blank TrichoSol™ or TrichoFoam™ solution, and a blank solution of the mobile phase/diluents, both with and without the matrix. The acceptance criterion for specificity was set as a percentage discrepancy between peak areas below 2%. All analyses were performed in triplicate.

To ensure precision, the study included assessments of repeatability and intermediate precision. Repeatability was evaluated by one analyst performing six replicates of consecutive analysis in a single day. Intermediate precision was evaluated by different analysts performing six replicates on two different days. An injection precision with a coefficient of variation below 5% was considered appropriate.

Accuracy measurements were conducted by the same analyst injecting chromatographic samples with the matrix added at concentration levels used in the linearity test (*n* = 3 for each concentration level). The results were expressed as a percentage of recovery compared to the analytical curve obtained from linearity.

For the linearity test, three standard curves were constructed using the API concentrations listed in Tables **5** and **6**, to assess the relationship between the analyte's concentration and the corresponding peak areas. The data for each concentration range were subjected to analysis of variance (ANOVA) and evaluated using the least-squares method to determine the correlation coefficient of the calibration curve.

The limit of detection (LOD) and the limit of quantification (LOQ) were determined from three standard calibration curves and were calculated as shown in Equations (1) and (2), respectively:


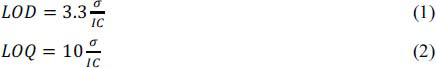


where IC is the mean slope of the analytical curves and σ is the standard deviation obtained from the noise estimate from the analysis of white samples (at least 10).

### 2.5 Preparation of Samples for the Compatibility Studies

The samples for the compatibility study were prepared using the following procedures. In some formulations, ethoxy diglycol was added to solubilize the API, levigating it before incorporating it into the vehicle. Sodium thiosulfate was also added in some formulations to improve photostability.

***F1. Minoxidil + 17-Alpha Estradiol (1.0% + 0.025% and 7.0% + 0.05%) in TrichoSol™/TrichoFoam™***

1. Calculate and weigh the required amount of each ingredient in the formulation.
2. Solubilize 17-alpha estradiol in ethoxy diglycol.
3. In another recipient, solubilize minoxidil in 1/3 of the total volume of TrichoSol™/TrichoFoam™ and mix until a clear mixture is obtained.
4. Add step 2 to step 3 and mix until homogeneous.
5. Solubilize sodium thiosulfate in a minimum amount of purified water and add to the previous step.
6. Bring to final volume with TrichoSol™/TrichoFoam™ stepwise.
7. Adjust the pH to 4.0-5.0 with triethanolamine if needed.

***F2. Minoxidil + Clobetasol (1.0 + 0.01% and 7.0% + 0.05%) in TrichoSol™/TrichoFoam™***

1. Calculate and weigh the required amount of each ingredient in the formulation.
2. Solubilize clobetasol in ethoxy diglycol.
3. In another recipient, solubilize minoxidil in 1/3 of the total volume of TrichoSol™/TrichoFoam™ and mix until a clear mixture is obtained.
4. Add step 2 to step 3 and mix until homogeneous.
5. Solubilize sodium thiosulfate in a minimum amount of purified water and add to the previous step.
6. Bring to final volume with TrichoSol™/TrichoFoam™ stepwise.
7. Adjust the pH to 4.0-5.0 with triethanolamine if needed.

***F3. Minoxidil + Dutasteride (5.0% + 0.1% and 7.0% + 0.25%) in TrichoSol™/TrichoFoam™***

1. Calculate and weigh the required amount of each ingredient in the formulation.
2. Grind dutasteride with sodium hyposulfite in a mortar and levigate with oleic acid.
3. Add ethoxy diglycol to the previous step and homogenize.
4. Add minoxidil to the previous step and homogenize.
5. Bring to final volume with TrichoSol™/TrichoFoam™ stepwise.
6. Adjust the pH to 4.0-5.0 with triethanolamine if needed.

***F4. Minoxidil + Finasteride (1.0% + 0.1% and 7.0% + 0.25%) in TrichoSol™/TrichoFoam™***

1. Calculate and weigh the required amount of each ingredient in the formulation.
2. Solubilize finasteride in oleic acid, add ethoxy diglycol and homogenize.
3. Solubilize minoxidil and sodium benzoate in 1/3 of the total volume of TrichoSol™/TrichoFoam™.
4. Pour step 2 into step 3 under constant mixing.
5. Bring to final volume with TrichoSol™/TrichoFoam™ stepwise.
6. Adjust the pH to 4.0-5.0 with triethanolamine if needed.

***F5. Minoxidil + Ketoconazole (1.0% + 0.5% and 7.0% + 2.0%) in TrichoSol™/TrichoFoam™***

1. Calculate and weigh the required amount of each ingredient in the formulation.
2. Grind minoxidil, ketoconazole and sodium metabisulfite in a mortar.
3. Solubilize in 1/3 of the total volume of TrichoSol™/TrichoFoam™ and mix until homogeneous.
4. Bring to final volume with TrichoSol™/TrichoFoam™ stepwise.
5. Adjust the pH to 4.0-5.0 with triethanolamine, if needed.

After compounding, formulations were stored in amber glass bottles at controlled room temperature (15-25 ºC) until the study was completed.

### 2.6 Compatibility Study: Physico-Chemical Evaluation

The APIs samples were assayed by HPLC at pre-determined time points to verify the stability of the APIs in TrichoSol™. Before analysis, the bottles were shaken for 1 minute to ensure uniform redispersion of suspended APIs. Aliquots for quantification (variable for each API) were withdrawn and diluted to obtain work solutions in the concentration described in Table **1**. Sampling times were: 0 days (T = 0), 7 days (T = 7), 14 days (T = 14), 30 days (T = 30), 60 days (T = 60), 90 days (T = 90), 120 days (T = 120), 150 days (T = 150) and 180 days (T = 180). The pH was also measured in all sampling times, and a visual inspection was performed to check for phase separation, sedimentation, and discoloration.

All solutions were assayed six times, and the results were expressed as the mean from six independent measurements. For that purpose, samples were diluted, sonicated for 10 minutes, and then filtered in 15-mm regenerated cellulose syringe filters with 0.45-μm pore size before injection into the HPLC system. The evaluation parameter was the percent recovery with respect to T = 0, using the HPLC method (results given as percentage ± standard deviation).

### 2.7 Compatibility Study: Microbiological Evaluation

The samples were analyzed for Antimicrobial Effectiveness Testing (AET) in 0 and 180 days after compounding, following the general USP <51> chapter [41]. The aliquots were withdrawn from the initial product and diluted to obtain working solutions. The microorganisms used in the AET were: *Candida albicans*, ATCC 10231; *Aspergillus brasiliensis*, ATCC 16404; *Escherichia coli*, ATCC 8739; *Pseudomonas aeruginosa*, ATCC 9027; *Staphylococcus aureus*, ATCC 6538.

A suspension of microorganisms was prepared and standardized on an optical scale at a concentration equivalent to 10^8^ colony-forming units (CFU)/mL; afterward, the suspension was inoculated in the sample respecting the range of 0.5% and 1.0% in relation to the weight of the total product.

A neutralizing agent (polysorbate and lecithin) was added to the sample prepared for plating dilution. The depth plating method determined the number of CFUs in the sample at the initial time (0 hours) and at each required time interval (14 and 28 days). The analyses were performed at T = 0 and T = 180 of the physical-chemical study.

## 3 RESULTS

Stability studies were conducted to assess the validity and adequacy of the chromatographic analysis methods used to identify the decomposition of the APIs. The decomposition profiles of the APIs, as shown in Tables **3** and **4**, exhibited similar trends when subjected to different stress conditions, including acidic, alkaline, heat, and UV light stresses. Negative values indicate how much the API was degraded by the given condition. Positive values may indicate the possible formation of extra compounds that elute together with the main API.

Following the determination of the forced degradation profiles, the stability of the APIs in TrichoSol™ and TrichoFoam™ was evaluated. The validation of all analysis methods was performed, and the results, presented in Tables **5** and **6**, met the respective acceptance criteria, thereby confirming the suitability of these methods for the intended objectives of this study.

The visual appearance of the topical solutions was evaluated at each sampling time to assess the physical stability and homogeneity. Throughout the study, no phenomena such as phase separation, sedimentation, or flocculation were observed when the drug content complied with the specified requirements.

Chemical stability results, expressed as the relative percentage of recovery compared to the initial sampling time (100%), are presented in Tables **7** and **8**. Additionally, Figs. (**1** and **2**) provide the absolute amounts of the APIs. For the formulations to be considered stable, the relative percentage of recovery should fall within 90% to 110% of the labeled amount.

The formulations underwent microbiological stability testing to evaluate their safety and product quality throughout processing and storage. The results indicated that after 14 and 28 days, the number of microorganisms in all formulations remained below the acceptable limit for the entire evaluation period. There was no increased bacterial count, and significant reductions in initially inoculated CFUs were observed.

## 4 DISCUSSION

In our research, we adopted the bracketing design strategy as advised by the ICH guidelines for stability testing of drug substances and products [42]. By doing so, we were able to simplify the stability study procedure and reduce the number of samples examined, thereby enhancing the efficiency of resource allocation.

It is important to highlight that our investigation centered on formulations compounded with a particular vehicle line (namely TrichoSol™ and TrichoFoam™) and within specified concentration ranges. As a result, the findings derived from this study pertained solely to the tested formulations and should not be generalized to other concentration ranges or alternative vehicles. To ensure dependable stability assessment under such circumstances, conducting supplementary studies covering a broader range of concentrations and diverse vehicles is crucial.

By following the ICH guidelines and employing a bracketing design methodology, we have acquired significant insights into the stability attributes of various APIs. This understanding aids in informed decision-making during pharmaceutical formulation development, facilitating product stability and quality enhancement across personalized compounded preparations.

The microbiological assessment in this research was conducted following the USP <51> Standard guidelines [41]. The AET was performed to evaluate the efficacy of the preservative system. The formulation samples were intentionally tainted with various microorganisms, including Gram-positive and Gram-negative bacteria, bacilli bacteria, yeast, and mold. Each microorganism was introduced into a distinct sample for precise monitoring purposes. Subsequently, the samples were monitored for microbial proliferation after intervals of 14 and 28 days. For the preservative system to be deemed efficient, it had to exhibit control over microbial proliferation by satisfying predetermined threshold criteria.

Previous studies have demonstrated the compatibility of minoxidil, clobetasol, dutasteride, and finasteride, separately, in TrichoSol™ and TrichoFoam™ [21-23]. However, until now, physicochemical evaluation of associated APIs has not been performed. Additionally, literature data to act as a comparative factor for our findings is scarce, which enhances the importance of our research. Nevertheless, some data can be used as a basic comparison to our findings.

The stability of minoxidil in compounded topical formulations has been largely studied throughout the years. In most cases, products remained stable for up to 180 days, except where the study perdured for shorter periods [43-45]. Previous studies have evaluated the stability of clobetasol associated with tacrolimus ointments, and no significant drug degradation was observed after 28 days [46]. Moreover, a study evaluating the stability of a clobetasol-loaded nanoemulsion showed no statistical changes in the formulation after 90 days [47]. This highlights the relevance of the data presented by this study, which demonstrates that minoxidil combined with clobetasol remained stable for up to 180 days in TrichoSol™, and 120 days in TrichoFoam™.

Finasteride is particularly sensitive concerning stability. A study on the development of a liposomal finasteride system has shown that, under refrigerated conditions, the formulation remained stable for 60 days [48]. In another study, iron oxide nanoparticles were loaded with finasteride or dutasteride, and their stability was evaluated over 90 days, showing a high stability profile at pH 7.4 [49]. Furthermore, dutasteride formulated as nanoemulsions in transdermal patches had its stability evaluated for 90 days at both room temperature and refrigerated, and no statistically significant changes were observed [50]. This data is in line with the results observed in our study, where the formulations containing minoxidil combined with finasteride remained stable for up to 90 days in TrichoSol™ and up to 60 days in TrichoFoam™. Minoxidil combined with dutasteride remained stable for up to 120 days in TrichoSol™ and up to 180 days in TrichoFoam™. It is relevant to enhance that this study evaluated a combination of APIs, which can potentially increase stability challenges to aqueous formulations.

Ketoconazole has been widely used in topical formulations targeting dermatological conditions. A study from Skiba *et al*. evaluated the stability of ketoconazole in aqueous formulations and found that formulations containing 1.0% of butylated hydroxytoluene (BHT) were stable for 6 months at pH 7.0 and that this was dependent on the concentration of BHT and of the pH [51]. In our research, formulations containing minoxidil combined with ketoconazole were stable for up to 150 days in TrichoSol™ and up to 120 days in TrichoFoam™.

According to our research and from the available data presented above, there is no data on the stability of the listed APIs in solutions or foams except for ketoconazole. To the best of our knowledge, there are no studies regarding the stability of 17-alpha estradiol in aqueous formulations. This cements the clinical relevance of our data to healthcare providers in personalized medicine by aiding potential therapeutic alternatives for patients undergoing alopecia treatments. We also highlight the need for further studies including microscopy, infrared spectroscopy, and differential scanning calorimetry to support the data presented here.

## CONCLUSION

Following comprehensive microbiological and physicochemical evaluations, the beyond-use date (BUD) that can be established for the formulations compounded in TrichoSol™, stored at room temperature, can be described as:

- F1: Minoxidil + 17-Alpha Estradiol, 1.0% + 0.025% and 7.0% + 0.05%, BUD 180 days;
- F2: Minoxidil + Clobetasol, 1.0% + 0.01% and 7.0% + 0.05%, BUD 180 days;
- F3: Minoxidil + Dutasteride, 5.0% + 0.1% and 7.0% + 0.25%, BUD 120 days;
- F4: Minoxidil + Finasteride, 1.0% + 0.1% and 7.0% + 0.25%, BUD 90 days;
- F5: Minoxidil + Ketoconazole, 1.0% + 0.5% and 7.0% + 2.0%, BUD 150 days; and

The BUD that can be established for the formulations compounded in TrichoFoam™, stored at room temperature, can be described as:

- F1: Minoxidil + 17-Alpha Estradiol, 1.0% + 0.025% and 7.0% + 0.05%, BUD 180 days;
- F2: Minoxidil + Clobetasol, 1.0% + 0.01%, BUD 120 days, and 7.0% + 0.05%, BUD 150 days;
- F3: Minoxidil + Dutasteride, 5.0% + 0.1% and 7.0% + 0.25%, BUD 180 days;
- F4: Minoxidil + Finasteride, 1.0% + 0.1%, BUD 60 days, and 7.0% + 0.25%, BUD 90 days;
- F5: Minoxidil + Ketoconazole, 1.0% + 0.5%, BUD 120 days, and 7.0% + 2.0%, BUD 150 days.

These findings emphasize the importance of ongoing stability studies and quality control measures to maintain the integrity and efficacy of the products. In conclusion, we can suggest that the vehicles TrichoSol™ and TrichoFoam™ present an effective solution for personalized hair care treatments.

## Figures and Tables

**Fig. (1):**
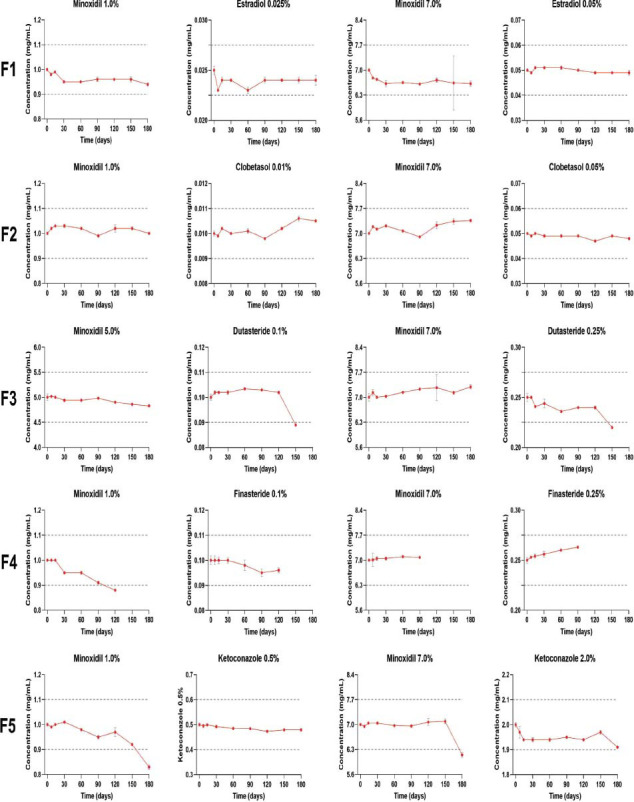
Active pharmaceutical ingredients’ chemical stability in TrichoSol™, in absolute amounts, measured by high-performance liquid chromatography.

**Fig. (2):**
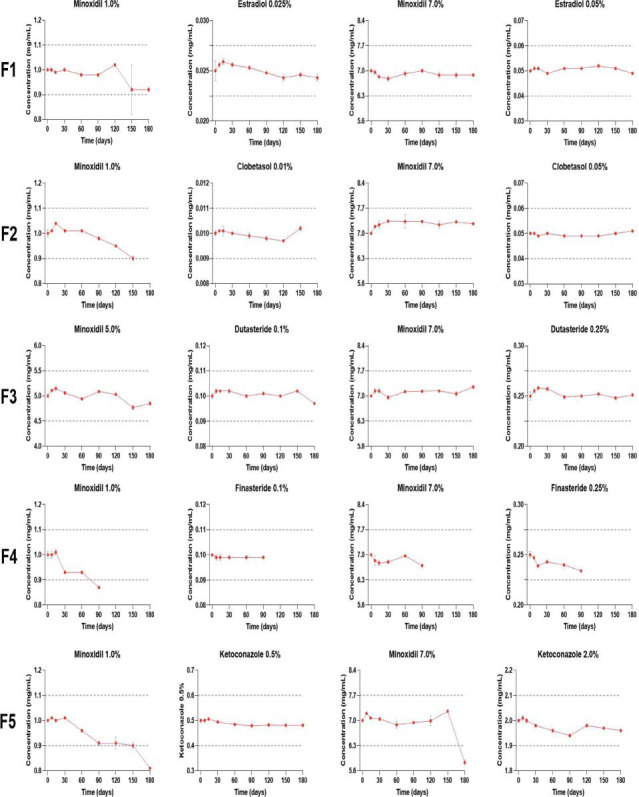
Active pharmaceutical ingredients’ chemical stability in TrichoFoam™, in absolute amounts, measured by high-performance liquid chromatography.

**Table 1:** APIs were tested for their compatibility with TrichoSol™ and TrichoFoam™ in the bracketed study.

**Active Pharmaceutical** **Ingredient**	**Concentration (%)**	Pharmaceutical Indication*
17-alpha estradiol	0.025 to 0.05	Low-potency form of estrogen, which acts as a 5-alpha reductase inhibitor, is used topically for androgenetic alopecia treatments.
Clobetasol	0.01 to 0.05	High-potency corticosteroids are used topically to treat various skin and scalp conditions, such as plaque psoriasis and eczema.
Dutasteride	0.1 to 0.25	5-alpha reductase (types 1 and 2) inhibitor and its topical use has been investigated for alopecia treatments.
Finasteride	0.1 to 0.25	5-alpha reductase (type 2) with anti-androgenic properties used to treat alopecia in men.
Ketoconazole	0.5 to 2.0	Imidazole antifungal is used topically to treat fungal infections of the skin and scalp, such as dandruff, seborrheic dermatitis, and pityriasis versicolor.
Minoxidil	1.0 to 7.0	Vasodilator drug widely used topically in alopecia treatments for increasing hair thickness and growth.

**Note:** [34, 35].

**Table 2:** Chromatographic conditions used in the compatibility study of different APIs with TrichoSol™ and TrichoFoam™.

**Active** **Pharmaceutical** **Ingredient**	**Mobile Phase** **Composition**	**Working** **Concentration** **(µg/mL)**	**Column**	**Flux** **(mL/min)**	**Ultraviolet** **Detection** **Wavelength (nm)**
17-alpha estradiol	Acetonitrile, water (550:450, v/v)	25, in methanol; 0.5 µL injection	L1 (C18), 2.1 x 50 mm, 25 °C	0.3	205
Clobetasol	0.01 M monobasic sodium phosphate buffer pH 2.5, acetonitrile, methanol (85:95:25, v/v/v)	20, in methanol; 0.5 µL injection	L1 (C18), 2.1 x 50 mm, 25 °C	0.4	240
Dutasteride	Methanol, water, acetic acid (7:3:0.1, v/v/v)	100, in acetonitrile in water (6:4, v/v) 0.5 µL injection	L1 (C18), 2.1 x 50 mm, 50 °C	0.3	237
Finasteride	2.5 mM Phosphoric acid, acetonitrile (100:100, v/v)	50, in acetonitrile in water (700:300, v/v); 20 µL injection	L1 (C18), 4.6 x 100 mm, 40 °C	1.0	240
Ketoconazole	SA + SB (7:3, v/v) SA: 2.8 mL triethanolamine in 700 mL methanol; SB: 600 mg ammonium acetate in 300 mL water. pH adjusted to 5.0 with acetic acid	100, in mobile phase; 1.0 µL injection	L1 (C18), 2.1 x 100 mm, 25 °C	0.3	225
Minoxidil (in combination with Finasteride)	Methanol, water, acetic acid (700:300:10, v/v/v)	100, in ethanol; 20 µL injection	L1 (C18), 4.6 x 250 mm, 25 °C	0.5	254
Minoxidil (remaining combinations)	SA + SB (Gradient*) SA: methanol, water, acetic acid (7:3:1, v/v/v); SB: 1.0 mL acetic acid in 1 L purified water	100, in SA; 0.5 µL injection	L1 (C18), 2.1 x 50 mm, 50 °C	0.4	280

**Note:** v = volume; SA = Solution A; SB = Solution B.

**Table 3:** Summary of the study indicating the stability of the APIs in TrichoSol™.

**Formulation**	**Active Pharmaceutical** **Ingredient**	**HCl** **(%d)**	**NaOH** **(%d)**	**UV** **(%d)**	**Heat** **(%d)**	**H_2_O_2_** **(%d)**
F1	Minoxidil 17-Alpha Estradiol	-49.32 -9.08	-38.07 -95.92	-3.22 -11.83	-6.79 -1.96	-39.18 -4.96
F2	Minoxidil Dutasteride	-49.37 -18.86	-41.03 -19.32	-7.84 -66.99	-11.24 -38.86	-42.08 -11.25
F3	Minoxidil Clobetasol	-51.36 -5.33	-42.05 -98.89	-9.44 10.97	-12.78 -9.67	-43.10 -10.80
F4	Minoxidil Finasteride	-26.15 -9.81	28.45 89.64	-21.43 -19.63	-20.33 -7.83	-19.81 -37.99
F5	Minoxidil Ketoconazole	-49.88 -82.05	-42.20 -26.65	-9.67 -7.20	-13.00 -7.14	-43.23 -16.59

**Abbreviations:** API, active pharmaceutical ingredient; HCl, hydrochloride acid; NaOH, sodium hydroxide solution; UV, ultraviolet; H_2_O_2_, peroxide. The results are presented as the average of six replicates at the working concentration. %d, the percentage of the discrepancy between the active pharmaceutical ingredient peak without stress factors (negative control) and the peak of a sample subjected to one of the accelerated degradation factors. A discrepancy of less than 2% indicates non-significant degradation of the API.

**Table 4:** Summary of the study indicating the stability of the APIs in TrichoFoam™.

**Formulation**	**Active Pharmaceutical** **Ingredient**	**HCl** **(%d)**	**NaOH** **(%d)**	**UV** **(%d)**	**Heat** **(%d)**	**H_2_O_2_** **(%d)**
F1	Minoxidil 17-Alpha Estradiol	-60.63 -2.76	-2.13 -99.93	2.91 4.49	-3.06 -0.58	-70.06 -3.30
F2	Minoxidil Dutasteride	-60.42 -9.88	-6.21 -9.33	-1.38 -66.73	-7.10 -27.06	-71.31 -14.04
F3	Minoxidil Clobetasol	-62.50 8.23	-7.78 -95.81	-3.04 -8.35	-8.66 20.33	-71.79 4.63
F4	Minoxidil Finasteride	-18.14 -21.56	103.55 -61.79	-12.46 -8.22	-2.79 -19.65	-39.29 -17.75
F5	Minoxidil Ketoconazole	-60.28 -62.95	-6.05 -31.02	-1.22 -0.13	-6.94 2.60	-71.26 -10.01

**Abbreviations:** API, active pharmaceutical ingredient; HCl, hydrochloride acid; NaOH, sodium hydroxide solution; UV, ultraviolet; H2O2, peroxide. The results are presented as the average of six replicates at the working concentration. %d, the percentage of the discrepancy between the active pharmaceutical ingredient peak without stress factors (negative control) and the peak of a sample subjected to one of the accelerated degradation factors. A discrepancy of less than 2% indicates non-significant degradation of the API.

**Table 5:** Summary of the validation results of the HPLC methods for formulations in TrichoSol™.

**-**	**Active** **Pharmaceutical** **Ingredient**	**Linearity**						**Specificity**	**Precision**		**Accuracy**
		**Range** **(µg/mL)**	**Analytical Curve**	**R^2^**	**ANOVA** **Significance of** **Regression (F)**	**LOD** **(µg/mL)**	**LOQ** **(µg/mL)**	**Discrepancy (%)**	**Repeatability (cv, %)**	**Intermediate** **Precision** **(cv, %)**	**Recovery (%)**
F1	Minoxidil 17-Alpha Estradiol	69.44-129.96 17.92-33.28	y = 0.1317x+0.8684 y = 0.1628x−0.1784	0.999 0.993	6609.01 867.90	0.06 0.38	0.18 1.16	0.31 1.70	1.38 0.39	3.27 0.52	100.08 100.17
F2	Minoxidil Dutasteride	69.44-128.96 69.44-128.96	y = 0.1317+0.8684 y = 0.0263x+0.172	0.999 0.992	6609.01 779.54	0.14 0.12	0.43 0.35	1.67 0.84	1.07 4.38	4.05 3.24	100.08 101.58
F3	Minoxidil Clobetasol	69.44-128.96 15.26-28.24	y = 0.1317x+0.8684 y = 0.041x+0.0318	0.999 0.990	6609.01 649.50	0.14 0.05	0.43 0.15	1.88 1.84	1.07 0.25	4.05 4.27	100.08 100.79
F4	Minoxidil Finasteride	72.10-133.90 35.49-65.91	y = 92.581x−1145.1 y = 14.516x−103.36	0.999 0.994	13741.43 1094.91	0.07 6.59	0.21 19.96	1.87 1.85	0.23 1.28	1.88 4.32	100.24 100.06
F5	Minoxidil Ketoconazole	69.44-128.96 72.52-134.68	y = 0.1317x+0.8684 y = 0.1166x+0.07	0.999 0.997	6609.01 1970.16	0.14 0.05	0.43 0.15	1.93 -1.96	1.07 2.15	4.05 1.32	100.08 99.36

**Abbreviations:** HPLC, high-performance liquid chromatography; ANOVA, Analysis of variance; CV, coefficient of variation; LOD, limit of detection; LOQ, limit of quantification. The acceptance criteria were: R2 > 0.99; F (significance of regression) >> 4.67; discrepancy < 2%; repeatability and intermediate precision < 5%; and recovery = 100% ± 2%. All analytical ranges were considered adequate to analyze the concentrations used.

**Table 6:** Summary of the validation results of the HPLC methods for formulations in TrichoFoam™.

**-**	**Active** **Pharmaceutical** **Ingredient**	**Linearity**						**Specificity**	**Precision**		**Accuracy**
		**Range** **(µg/mL)**	**Analytical Curve**	**R^2^**	**ANOVA** **Significance of** **Regression (F)**	**LOD** **(µg/mL)**	**LOQ** **(µg/mL)**	**Discrepancy (%)**	**Repeatability (cv, %)**	**Intermediate** **Precision** **(cv, %)**	**Recovery (%)**
F1	Minoxidil 17-Alpha Estradiol	69.44-128.96 17.92-33.28	y = 0.1317x+0.8684 y = 0.1628x-0.1784	0.999 0.993	6609.01 867.9	0.06 0.17	0.18 0.51	-1.19 1.86	1.38 0.44	3.27 3.88	100.08 100.17
F2	Minoxidil Dutasteride	69.44-128.96 69.44-128.96	y = 0.1317x+0.8684 y = 0.0263x+0.172	0.999 0.991	6609.01 779.54	0.06 0.35	0.18 1.06	1.67 0.36	1.38 1.36	3.27 3.96	100.08 101.58
F3	Minoxidil Clobetasol	69.44-128.96 15.26-28.34	y = 0.1317x+0.8684 y = 0.041x+0.0318	0.999 0.991	6609.01 649.50	0.06 0.04	0.18 0.1	-1.84 0.92	1.38 0.19	3.27 4.83	100.08 100.79
F4	Minoxidil Finasteride	72.10-133.90 35.49-65.91	y = 92.581x−1145.1 y = 14.516x−103.36	0.999 0.994	13741.43 1094.91	0.07 5.58	0.21 16.90	0.44 1.71	0.23 1.47	1.88 2.53	100.24 100.06
F5	Minoxidil Ketoconazole	69.44-128.96 72.52-134.68	y = 0.1317x+0.8684 y = 0.1166x+0.07	0.999 0.997	6609.01 1970.16	0.06 0.01	0.18 0.03	1.60 1.68	1.38 1.78	3.27 4.17	100.08 99.36

**Abbreviations:** HPLC, high-performance liquid chromatography; ANOVA, Analysis of variance; CV, coefficient of variation; LOD, limit of detection; LOQ, limit of quantification. The acceptance criteria were: R2 > 0.99; F (significance of regression) >> 4.67; discrepancy < 2%; repeatability and intermediate precision < 5%; and recovery = 100% ± 2%. All analytical ranges were considered adequate to analyze the concentrations used.

**Table 7:** API's chemical stability in TrichoSol^TM^, measured by HPLC.

**Formula**	**Active Pharmaceutical** **Ingredients**	**Elapsed Time (Days)**		**% Recovery (Room Temperature, 15-30 °C)**									
F1	Minoxidil + 17-Alpha Estradiol (1.0% + 0.025% and 7.0% + 0.05%)	**-**		**Low Concentration**						**High Concentration**			
		**-**		**Minoxidil**		**17-Alpha** **Estradiol**		**pH**		**Minoxidil**		**17-Alpha** **Estradiol**	**pH**
		0		100.00 ± 0.54		100.00 ± 1.51		3.02		100.00 ± 0.16		100.00 ± 0.44	4.00
		7		98.35 ± 0.64		93.22 ± 0.29		3.04		96.87 ± 0.15		98.61 ± 0.17	4.02
		14		98.85 ± 0.44		94.98 ± 1.18		3.04		96.34 ± 0.55		101.00 ± 0.86	4.00
		30		95.33 ± 0.20		94.51 ± 0.37		3.01		94.54 ± 1.24		102.19 ± 0.75	4.04
		60		95.24 ± 0.26		93.84 ± 1.23		2.90		95.04 ± 0.28		101.24 ± 1.40	3.97
		90		96.38 ± 1.03		95.40 ± 1.09		2.97		94.44 ± 0.43		99.89 ± 0.28	4.01
		120		95.58 ± 0.25		95.24 ± 0.44		3.03		96.03 ± 0.90		98.99 ± 0.14	4.05
		150		95.51 ± 1.08		94.56 ± 1.31		2.95		94.81 ± 1.09		98.66 ± 0.64	4.05
		180		93.59 ± 0.67		95.23 ± 1.81		3.09		94.63 ± 1.03		98.10 ± 1.91	4.04
F2	Minoxidil + Clobetasol (1.0 + 0.01% and 7.0% + 0.05%)	**-**		**Low Concentration**						**High Concentration**			
		**-**		**Minoxidil**		**Clobetasol**		**pH**		**Minoxidil**		**Clobetasol**	**pH**
		0		100.00 ± 0.60		100.00 ± 0.60		2.84		100.00 ± 0.41		100.00 ± 0.22	3.99
		7		102.50 ± 0.35		98.97 ± 0.19		2.89		102.73 ± 0.30		97.38 ± 0.30	3.90
		14		103.19 ± 0.29		101.94 ± 0.21		2.85		101.74 ± 0.28		99.52 ± 026	3.92
		30		103.09 ± 0.69		100.47 ± 0.33		3.02		103.04 ± 0.42		98.61 ± 0.45	3.99
		60		101.58 ± 0.43		101.21 ± 0.79		2.93		101.06 ± 0.21		97.80 ± 0.48	3.87
		90		99.42 ± 0.32		98.23 ± 0.10		2.91		98.60 ± 0.45		97.91 ± 0.57	3.89
		120		101.83 ± 1.50		102.20 ± 0.28		2.93		103.36 ± 1.40		94.86 ± 0.57	3.83
		150		101.98 ± 067		105.95 ± 0.55		3.01		104.79 ± 1.18		97.74 ± 0.20	3.88
		180		99.92 ± 0.34		104.93 ± 0.49		2.94		105.15 ± 0.47		96.15 ± 0.50	3.91
F3	Minoxidil + Dutasteride (5.0% + 0.1% and 7.0% + 0.25%)	**-**		**Low Concentration**						**High Concentration**			
		**-**		**Minoxidil**		**Clobetasol**		**pH**		**Minoxidil**		**Clobetasol**	**pH**
		0		100.00 ± 1.31		100.00 ± 1.16		3.65		100.00 ± 1.54	100.00 ± 1.73		3.88
		7		100.44 ± 0.40		102.34 ± 0.74		3.60		100.82 ± 1.26	99.86 ± 0.24		3.84
		14		100.06 ± 0.17		102.28 ± 0.42		3.58		99.97 ± 0.39	96.46 ± 0.12		3.84
		30		99.79 ± 0.21		101.83 ± 0.76		3.61		100.38 ± 0.26	97.59 ± 1.75		3.84
		60		98.86 ± 0.21		103.53 ± 0.55		3.60		102.02 ± 0.35	94.21 ± 0.22		3.86
		90		99.63 ± 0.19		103.15 ± 0.34		3.70		103.29 ± 0.21	95.88 ± 0.21		3.91
		120		98.08 ± 0.19		102.09 ± 0.60		3.65		103.81 ± 0.52	96.07 ± 0.62		3.82
		150		97.12 ± 0.25		89.33 ± 0.11*		3.59		101.89 ± 0.63	88.10 ± 0.02*		3.80
F4	Minoxidil + Finasteride (1.0% + 0.1% and 7.0% + 0.25%)	**-**		**Low Concentration**						**High Concentration**			
		**-**		**Minoxidil**		**Clobetasol**		**pH**		**Minoxidil**		**Clobetasol**	**pH**
		0		100.00 ± 0.47		100.00 ± 1.84		2.90		100.00 ± 0.11	100.00 ± 0.98		3.78
		7		100.06 ± 0.21		100.01 ± 1.84		2.94		100.17 ± 2.70	101.27 ± 0.46		3.82
		14		99.86 ± 0.40		99.52 ± 1.33		2.90		100.66 ± 0.81	101.70 ± 0.70		3.81
		30		95.14 ± 0.27		100.02 ± 1.05		2.78		100.71 ± 0.71	102.34 ± 1.07		3.70
		60		94.92 ± 0.62		98.12 ± 2.12		2.98		101.50 ± 0.42	104.08 ± 0.29		3.63
		90		90.87 ± 0.62		95.17 ± 1.47		2.90		101.16 ± 0.28	105.08 ± 0.29		3.70
		120		88.15 ± 0.28**		95.66 ± 0.82		2.92		***	***		-
F5	Minoxidil + Ketoconazole (1.0% + 0.5% and 7.0% + 2.0%)	**-**	**Low Concentration**						**High Concentration**				
		**-**	**Minoxidil**		**Ketoconazole**		**pH**		**Minoxidil**		**Ketoconazole**		**pH**
		0	100.00 ± 0.66		100.00 ± 1.11		2.89		100.00 ± 0.24		100.00 ± 0.39		3.95
		7	99.25 ± 0.35		99.08 ± 1.45		2.76		99.34 ± 0.24		98.61 ± 1.14		3.94
		14	99.87 ± 0.34		99.73 ± 0.28		2.80		100.63 ± 0.57		96.85 ± 0.13		3.89
		30	100.77 ± 0.25		98.42 ± 0.23		2.92		100.59 ± 0.20		96.92 ± 0.39		3.85
		60	97.91 ± 0.23		96.94 ± 0.44		2.97		99.61 ± 0.35		96.80 ± 0.38		4.01
		90	95.23 ± 0.68		96.71 ± 0.61		2.83		99.40 ± 0.18		97.52 ± 0.12		3.84
		120	96.21 ± 1.83		94.57 ± 0.12		2.83		101.06 ± 1.47		96.80 ± 0.13		3.85
		150	91.94 ± 0.22		95.82 ± 0.11		2.85		101.34 ± 1.00		98.66 ± 0.40		3.80
		180	83.15 ± 0.96**		95.78 ± 0.14		2.90		87.90 ± 1.06**		95.46 ± 0.41		3.87

**Note:** * The dutasteride contents were below the minimum acceptable limit for the specification at 150 days for both concentrations, making it impossible to continue the study. ** The minoxidil contents were below the minimum acceptable limit for the specification at 180 days. *** Sample with the formation of precipitates, making the analysis impossible to be performed.

**Table 8:** API's chemical stability in TrichoFoam^TM^, measured by HPLC.

**Formula**	**Active Pharmaceutical** **Ingredients**		**Elapsed Time (Days)**		**% Recovery (Room Temperature, 15-30 °C)**									
F1	Minoxidil + 17-Alpha Estradiol (1.0% + 0.025% and 7.0% + 0.05%)		**-**		**Low Concentration**						**High Concentration**			
			**-**		**Minoxidil**		**17-Alpha** **Estradiol**		**pH**		**Minoxidil**		**17- Alpha Estradiol**	**pH**
			0		100.00 ± 0.22		100.00 ± 0.50		2.76		100.00 ± 0.25		100.00 ± 0.88	3.78
			7		100.45 ± 0.92		102.20 ± 0.98		2.74		99.37 ± 0.83		102.23 ± 1.12	3.77
			14		98.58 ± 0.18		103.66 ± 1.22		2.76		97.55 ± 0.19		102.03 ± 1.22	3.78
			30		100.12 ± 0.18		102.47 ± 0.85		2.71		96.89 ± 1.01		98.65 ± 0.64	3.74
			60		98.00 ± 0.21		101.12 ± 0.72		2.85		98.91 ± 1.08		102.36 ± 0.95	3.81
			90		97.91 ± 0.26		99.32 ± 0.23		2.81		100.05 ± 0.61		102.64 ± 0.72	3.81
			120		101.51 ± 0.11		97.16 ± 1.38		2.86		98.22 ± 1.25		103.76 ± 0.93	3.81
			150		92.21 ± 1.00		98.38 ± 0.67		2.82		98.30 ± 1.13		101.11 ± 0.66	3.79
			180		91.89 ± 0.79		97.03 ± 1.10		2.86		97.56 ± 0.14		97.56 ± 0.14	3.81
F2	Minoxidil + Clobetasol (1.0% + 0.01% and 7.0% + 0.05%)		**-**		**Low Concentration**						**High Concentration**			
			**-**		**Minoxidil**		**Clobetasol**		**pH**		**Minoxidil**		**Clobetasol**	**pH**
			0		100.00 ± 1.49		100.00 ± 0.92		2.81		100.00 ± 0.19		100.00 ± 0.48	3.73
			7		100.74 ± 0.16		100.87 ± 0.31		2.74		102.65 ± 0.44		99.75 ± 0.23	3.64
			14		103.87 ± 0.30		100.52 ± 1.57		2.77		103.37 ± 1.58		98.56 ± 0.31	3.68
			30		101.27 ± 0.61		100.42 ± 0.36		2.84		104.91 ± 0.40		99.91 ± 0.38	3.84
			60		100.94 ± 0.30		99.34 ± 1.11		2.90		104.70 ± 0.28		98.40 ± 0.37	3.75
			90		97.66 ± 0.49		98.04 ± 0.56		2.74		104.72 ± 0.30		98.19 ± 0.31	3.64
			120		94.83 ± 0.27		97.33 ± 0.25		2.80		103.49 ± 1.58		98.87 ± 0.39	3.64
			150		89.83 ± 0.99*		101.90 ± 0.67		2.84		104.52 ± 0.50		99.35 ± 0.67	3.70
			180		-		-		-		103.90 ± 0.57		102.60 ± 0.72	3.70
F3	Minoxidil + Dutasteride (5.0% + 0.1% and 7.0% + 0.25%)		**-**		**Low Concentration**						**High Concentration**			
			**-**		**Minoxidil**		**Dutasteride**	**pH**		**Minoxidil**		**Dutasteride**		**-**
			0		100.00 ± 0.71	100.00 ± 1.03		3.43		100.00 ± 0.42		100.00 ± 0.71		3.64
			7		102.25 ± 0.48	102.29 ± 0.82		3.43		102.00 ± 0.94		101.96 ± 0.90		3.60
			14		103.25 ± 0.42	102.43 ± 0.41		3.40		102.05 ± 0.81		103.20 ± 0.30		3.58
			30		101.21 ± 0.60	102.22 ± 0.78		3.41		99.46 ± 0.68		102.62 ± 0.46		3.62
			60		98.72 ± 0.24	100.08 ± 0.58		3.49		101.70 ± 0.48		99.76 ± 0.75		3.62
			90		101.90 ± 0.46	100.78 ± 0.41		3.56		101.92 ± 0.34		99.80 ± 0.39		3.71
			120		100.54 ± 0.34	99.91 ± 0.49		3.47		101.98 ± 0.46		100.79 ± 0.48		3.61
			150		95.33 ± 0.74	102.49 ± 0.51		3.45		100.90 ± 0.86		99.37 ± 0.39		3.60
			180		97.10 ± 0.74	96.68 ± 0.51		3.44		103.56 ± 0.64		100.40 ± 0.39		3.70
F4	Minoxidil + Finasteride (1.0% + 0.1% and 7.0% + 0.25%)		**-**		**Low Concentration**						**High Concentration**			
			**-**		**Minoxidil**		**Finasteride**		**pH**		**Minoxidil**		**Finasteride**	**pH**
			0		100.00 ± 1.32		100.00 ± 0.17		2.79		100.00 ± 0.18		100.00 ± 1.29	3.74
			7		99.74 ± 1.38		98.55 ± 1.09		2.82		97.72 ± 1.99		98.93 ± 0.43	3.79
			14		101.13 ± 1.29		98.73 ± 1.34		2.83		96.77 ± 1.04		95.78 ± 0.16	3.78
			30		93.48 ± 0.12		98.95 ± 0.57		2.64		97.11 ± 0.07		97.09 ± 0.14	3.69
			60		93.04 ± 0.15		98.79 ± 0.56		2.66		99.61 ± 0.23		96.07 ± 0.59	3.62
			90		86.76 ± 0.18**		99.22 ± 0.38**		2.68		95.68 ± 0.29		93.80 ± 0.32	3.21
			120		-		-		-		**		**	-
F5	Minoxidil + Ketoconazole (1.0% + 0.5% and 7.0% + 2.0%)		**-**		**Low Concentration**						**High Concentration**			
			**-**		**Minoxidil**		**Ketoconazole**	**pH**		**Minoxidil**		**Ketoconazole**		**pH**
			0		100.00 ± 0.38		100.00 ± 1.54		2.45		100.00 ± 0.48		100.00 ± 0.19	3.94
			7		101.09 ± 0.47		100.08 ± 0.76		2.80		102.87 ± 0.32		100.60 ± 0.48	3.84
			14		100.34 ± 0.34		101.16 ± 0.27		2.85		101.07 ± 0.22		100.16 ± 0.42	3.90
			30		101.11 ± 0.30		98.77 ± 0.17		2.90		100.54 ± 0.70		99.17 ± 0.11	3.81
			60		95.58 ± 0.58		96.85 ± 0.09		2.99		98.29 ± 1.37		97.94 ± 0.33	3.82
			90		91.02 ± 0.79		95.81 ± 0.11		2.82		99.17 ± 0.12		97.00 ± 0.16	3.85
			120		90.81 ± 2.45		96.43 ± 0.14		2.77		99.79 ± 1.86		98.83 ± 0.17	3.82
			150		89.78 ± 1.24*		96.25 ± 0.20		2.84		103.74 ± 0.26		98.61 ± 0.23	3.79
			180		-		-		-		83.24 ± 1.05*		97.97 ± 0.11	3.84

**Note:** * The minoxidil contents were below the minimum acceptable limit for the specification at 180 days. ** Samples with the formation of precipitates, making the analysis impossible to be performed.

## Data Availability

All data generated or analyzed during this study are included in this published article.

## LIST OF ABBREVIATIONS

AET: Antimicrobial Effectiveness Testing
AGA: Androgenetic alopecia
ANOVA: Analysis of variance
API: Active Pharmaceutical Ingredient
ATCC: American Type Culture Collection
BHT: Butylated hydroxytoluene
BUD: Beyond-Use Date
CV: Coefficient of variation
FDA: Food and Drug Administration
H_2_O_2_: Peroxide
HCl: Hydrochloric acid
HPLC: High-performance liquid chromatography
ICH: International Council for Harmonization of Technical Requirements for Pharmaceuticals for Human Use
LOD: Limit of Detection
LOQ: Limit of Quantification
NaOH: Sodium hydroxide
USP: Unites States Pharmacopeia
UV: Ultraviolet

## References

[1] Jamerson T.A., Aguh C. (2021). An approach to patients with alopecia.. Med. Clin. North Am..

[2] Hunt N., McHale S. (2005). The psychological impact of alopecia.. BMJ.

[3] Yu L., Moorthy S., Peng L., Shen L., Han Y., Zhang Z., Li Y., Huang X. (2023). Evaluation of anxiety and depression in patients with androgenetic alopecia in Shanghai: A cross‐sectional study.. Dermatol. Ther..

[4] Yu N., Guo Y. (2024). Association between alopecia areata, anxiety, and depression: Insights from a bidirectional two-sample Mendelian randomization study.. J. Affect. Disord..

[5] Gordon K., Gordon K., Tosti A. (2011). Alopecia: Evaluation and treatment.. Clin. Cosmet. Investig. Dermatol..

[6] Lolli F., Pallotti F., Rossi A., Fortuna M.C., Caro G., Lenzi A., Sansone A., Lombardo F. (2017). Androgenetic alopecia: A review.. Endocrine.

[7] Mirmirani P., Khumalo N.P. (2014). Traction alopecia.. Dermatol. Clin..

[8] Pratt C.H., King L.E., Messenger A.G., Christiano A.M., Sundberg J.P. (2017). Alopecia areata.. Nat. Rev. Dis. Primers.

[9] Rahangdale P.C., Wankhade A.M. (2023). A review on-types and treatment of alopecia.. Asian J. Pharm. Res..

[10] Konisky H., Balazic E., Kobets K. (2024). Characterization of alopecia clinical trials: An analysis of trials registered on clinicaltrials.gov.. Arch. Dermatol. Res..

[11] Nestor M.S., Ablon G., Gade A., Han H., Fischer D.L. (2021). Treatment options for androgenetic alopecia: Efficacy, side effects, compliance, financial considerations, and ethics.. J. Cosmet. Dermatol..

[12] Goren A., Naccarato T. (2018). Minoxidil in the treatment of androgenetic alopecia.. Dermatol. Ther..

[13] Rambwawasvika H. (2021). Alopecia types, current and future treatment.. J. Cosmet. Dermatol..

[14] Shen Y., Zhu Y., Zhang L., Sun J., Xie B., Zhang H., Song X. (2023). New target for Minoxidil in the treatment of androgenetic alopecia.. Drug Des. Devel. Ther..

[15] Raval R.S., Nohria A., Desai D., Mourtzanakis K., Buontempo M., Shapiro J., Lo Sicco K. (2024). The use of minoxidil in the treatment of alopecia areata: A systematic review.. J. Am. Acad. Dermatol..

[16] Stoehr J.R., Choi J.N., Colavincenzo M., Vanderweil S. (2019). Off-label use of topical minoxidil in alopecia: A review.. Am. J. Clin. Dermatol..

[17] Suchonwanit P., Thammarucha S., Leerunyakul K. (2019). Minoxidil and its use in hair disorders: A review.. Drug Des. Devel. Ther..

[18] Friedman E.S., Friedman P.M., Cohen D.E., Washenik K. (2002). Allergic contact dermatitis to topical minoxidil solution: Etiology and treatment.. J. Am. Acad. Dermatol..

[19] Suneel R.S., Shivani V., Nikhil Kumar A., Amey K., Suyog M., Sujeet M., Charugulla N. (2020). Comparative study to evaluate tolerability of topical 5% Minoxidil novel formulation and alcohol-based conventional solutions in treatment of androgenetic alopecia in Indian men: Randomized double-blind Study.. Dermatol. Ther. (Heidelb.).

[20] Amaral F., Jardim M., de Souza Antunes V.M., Michelin L.F.G., dos Santos B.A.R., Barbosa C.M.V., Spindola D.G., Bincoletto C., Oliveira C.R. (2017). EEffects of the phytocomplex TrichoTechTM on human fibroblasts: Proliferative potential and effects on gene expression of FGF-7 and FGF-10.. J. Cosmet. Dermatol. Sci. Applic..

[21] Polonini H.C., Taylor S., Silva C.C.V. (2024). Compatibility of cetirizine hydrochloride, dutasteride, hydrocortisone acetate, nicotinamide, progesterone, and pyridoxine hydrochloride in TrichoSolTM, A natural vehicle for hair solutions.. Int. J. Pharm. Compd..

[22] Polonini H.C., Sousa P.L., Silva C.C.V. (2024). Compatibility of caffeine clobetasol propionate dutasteride nicotinamide and progesterone in TrichoFoam™, a natural vehicle for hair foams.. Int. J. Pharm. Compd..

[23] Polonini H., Taylor S., Zander C. (2022). Compatibility of different formulations in TrichoConceptTM vehicles for hair treatments.. Sci. Pharm..

[24] Polonini H.C., Silva C.C.V. (2023). Compounded hair solutions and foams containing Minoxidil: Does the color change impact stability?. Sci. Pharm..

[25] Panchaprateep R. (2024). Medical treatment for androgenetic alopecia.. Facial Plast. Surg..

[26] Kim J.H., Lee S.Y., Lee H.J., Yoon N.Y., Lee W.S. (2012). The efficacy and safety of 17α-Estradiol (Ell-Cranell® alpha 0.025%) solution on female pattern hair loss: Single center, open-label, non-comparative, phase iv study.. Ann. Dermatol..

[27] Molinelli E., Campanati A., Brisigotti V., Sapigni C., Paolinelli M., Offidani A. (2020). Efficacy and safety of topical calcipotriol 0.005% versus topical clobetasol 0.05% in the management of alopecia areata: An intrasubject pilot study.. Dermatol. Ther. (Heidelb.).

[28] Piraccini B.M., Blume-Peytavi U., Scarci F., Jansat J.M., Falqués M., Otero R., Tamarit M.L., Galván J., Tebbs V., Massana E. (2022). Efficacy and safety of topical finasteride spray solution for male androgenetic alopecia: A phase III, randomized, controlled clinical trial.. J. Eur. Acad. Dermatol. Venereol..

[29] Pindado-Ortega C., Saceda-Corralo D., Moreno-Arrones Ó.M., Rodrigues-Barata A.R., Hermosa-Gelbard Á., Jaén-Olasolo P., Vañó-Galván S. (2021). Effectiveness of dutasteride in a large series of patients with frontal fibrosing alopecia in real clinical practice.. J. Am. Acad. Dermatol..

[30] Ding Y., Wang C., Bi L., Du Y., Lu C., Zhao M., Fan W. (2024). Dutasteride for the treatment of androgenetic alopecia: An updated review.. Dermatology.

[31] Koralewicz M.M., Szatkowska O.A. (2024). Topical solutions for androgenetic alopecia: Evaluating efficacy and safety.. Forum Dermatologicum.

[32] Fields J.R., Vonu P.M., Monir R.L., Schoch J.J. (2020). Topical ketoconazole for the treatment of androgenetic alopecia: A systematic review.. Dermatol. Ther..

[33] (2024). U.S. Pharmacopeial Convention. Pharmaceutical compounding - Nonsterile preparations. In United States Pharmacopeia.. https://www.uspnf.com/sites/default/files/usp_pdf/EN/USPNF/revisions/gc-795-rb-notice-20200424.pdf.

[34] Drugs https://www.drugs.com/.

[35] Sweetman S.C. (2012). Martindale: The complete drug reference.. J. Med. Libr. Assoc..

[36] (2024). ICH Q1A (R2) stability testing of new drug substances and drug products: Scientific guideline.. https://database.ich.org/sites/default/files/Q1A%28R2%29%20Guideline.pdf.

[37] Polonini H., Marianni B., Taylor S., Zander C. (2022). Compatibility of personalized formulations in Cleoderm™, A skin rebalancing cream base for oily and sensitive skin.. Cosmetics.

[38] Guy R.C. (2014). International conference on harmonisation. Encyclopedia of Toxicology.

[39] (2005). Validation of analytical procedures: Text and methodology Q2(R1). https://database.ich.org/sites/default/files/Q2%28R1%29%20Guideline.pdf.

[40] Validation of microbial recovery from pharmacopeial articles. United States Pharmacopeia. https://doi.usp.org/USPNF/USPNF_M99947_03_01.html.

[41] (2024). Antimicrobial effectiveness testing. In USPNF.. https://doi.usp.org/USPNF/USPNF_M98790_03_01.html.

[42] European Medicines Agency ICH Q1D Bracketing and matrixing designs for stability testing of drug substances and drug products: Scientific guideline.. https://www.ema.europa.eu/en/ich-q1d-bracketing-matrixing-designs-stability-testing-drug-substances-drug-products-scientific.

[43] Geiger C.M., Sorenson B., Whaley P.A. (2013). Stability of minoxidil in Espumil foam base.. Int. J. Pharm. Compd..

[44] Lupatini R., Sidhu R., Patel H., Bichar K. (2021). Stability evaluation of minoxidil in FOAMIL foam base with bracketing study design.. Int. J. Pharm. Compd..

[45] Spennacchio A., Lopedota A.A., Lopalco A., Dibenedetto M.T., la Forgia F.M., Fontana S., Franco M., Denora N. (2023). Extemporaneous topical Minoxidil solutions for the treatment of alopecia: Stability studies and incorporation tests of active ingredients in ALOPLUS FAST base.. Int. J. Pharm. Compd..

[46] Pappas L., Kiss A., Levitt J. (2009). Compatibility of tacrolimus ointment with corticosteroid ointments of varying potencies.. J. Cutan. Med. Surg..

[47] Ali M.S., Alam M.S., Alam N., Anwer T., Safhi M.M. (2013). Accelerated stability testing of a clobetasol propionate loaded nanoemulsion as per ICH guideline.. Sci. Pharm..

[48] Kumar R., Singh B., Bakshi G., Katare O.P. (2007). Development of liposomal systems of finasteride for topical applications: Design, characterization, and in vitro evaluation.. Pharm. Dev. Technol..

[49] Afiune L.A.F., Ushirobira C.Y., Barbosa D.P.P., de Souza P.E.N., Leles M.I.G., Cunha-Filho M., Gelfuso G.M., Soler M.A.G., Gratieri T. (2020). Novel iron oxide nanocarriers loading finasteride or dutasteride: Enhanced skin penetration for topical treatment of alopecia.. Int. J. Pharm..

[50] Ali M.S., Alam M.S., Alam N., Siddiqui M.R. (2014). Preparation, characterization and stability study of dutasteride loaded nanoemulsion for treatment of benign prostatic hypertrophy.. Iran. J. Pharm. Res..

[51] Skiba M., Skiba-Lahiani M., Marchais H., Duclos R., Arnaud P. (2000). Stability assessment of ketoconazole in aqueous formulations.. Int. J. Pharm..

